# The Effect of Electropulsing-Assisted Ultrasonic Surface Rolling Process on the Friction and Wear Properties of AA 2024-T3

**DOI:** 10.3390/mi17060665

**Published:** 2026-05-28

**Authors:** Daoyin Xin, Guangjun Chen, Yuhang Liang, Wenqi Wang, Chuan He, Yingxin Lv, Bo Zhang, Jiashuai Huang

**Affiliations:** 1 School of Mechanical Engineering, Tianjin University of Technology and Education, Tianjin 300222, China; xdy_f@163.com (D.X.);; 2 Tianjin Key Laboratory of High Performance Manufacturing Technology and Equipment, Tianjin University of Technology and Education, Tianjin 300222, China; 3 School of Intelligent Engineering, Jiangsu Vocational College of Information Technology, Wuxi 214153, China; 4 School of Mechanical Engineering, Jiamusi University, Jiamusi 154007, China

**Keywords:** electropulsing-assisted ultrasonic surface rolling process (EP-USRP), AA 2024-T3, surface roughness, microhardness, friction and wear properties

## Abstract

Aluminum alloy (AA) 2024-T3 is widely used in the aerospace industry due to its high specific strength and superior fatigue resistance, yet its moderate hardness and inadequate wear resistance fail to meet service demands under severe wear conditions. Although the electropulsing-assisted ultrasonic surface rolling process (EP-USRP) can strengthen its surface, the mechanism by which pulsed current density influences the friction and wear properties of the strengthened layer remains unclear. This study employed EP-USRP to strengthen the surface properties of AA 2024-T3, systematically examining the impacts of different pulsed current densities on its surface morphology, roughness, microhardness, and tribological performance, while comparing wear mechanisms among differently treated samples under various normal loads. The results indicate that EP-USRP can improve the surface mechanical properties compared with USRP. At 1.42 A/mm^2^ current density, the EP-USRP-3 sample achieved 27.7% lower surface roughness and 19.6% higher microhardness. Under a 2 N normal load, its average friction coefficient, wear volume, and wear rate decreased by 5.34%, 36.28%, and 36.27%, respectively. Adhesive and oxidative wear, coupled with material spalling, constitute its main wear behavior. The electroplastic effect induced by pulsed current may facilitate the increase in strain levels and dislocation densities, which could contribute to the improved surface mechanical and tribological properties of AA 2024-T3.

## 1. Introduction

AA 2024-T3 is one of the most extensively applied and cost-effective alloys in the 2xxx series aluminum alloys, which demonstrates outstanding damage tolerance and excellent fatigue crack propagation resistance, particularly under the T3 temper state [1]. This material has been widely utilized in critical aerospace structural components, including the front pressure frame of Comac’s C919 of China and the rotating structures of the Japanese H-1 rocket [2]. According to the Vickers hardness test, AA 2024-T3 has a hardness of 122 HV. If no surface strengthening treatment is adopted, AA 2024-T3 exhibits moderate wear resistance compared with conventional aerospace aluminum alloys, rendering it incapable of servicing severe wear conditions and limiting its broader engineering applications. Therefore, surface strengthening technologies are essential to further enhance the hardness and wear resistance of AA 2024-T3.

Previous studies showed that the primary surface strengthening technologies for AA 2024-T3 include heat treatment [3,4], surface coating [5,6,7,8], shot peening [9,10,11], and laser shock peening [12]. Regrettably, these approaches come with limitations like complicated process flow, environmental pollution and high surface roughness [13,14,15]. The ultrasonic surface rolling process (USRP) is a recently developed surface severe plastic deformation (SPD) technique that integrates ultrasonic impact peening (UIP) and deep rolling (DR) [16]. Researchers have experimentally demonstrated that USRP can provide improved wear resistance [17,18,19,20], a reduction in surface roughness [21,22], remarkable increases in hardness [20,23], a significant amount of induced compressive residual stress (CRS) [21,22,24], superior grain refinement [21,25] and enhanced corrosion resistance [26,27]. Zhou et al. [28] fabricated gradient nanostructured aluminum alloys by means of a two-dimensional ultrasonic surface burnishing process (2D-USBP) and confirmed that this treatment can notably boost the wear resistance of aluminum alloys. However, in the later stage of USRP treatment, the significant strain hardening induced by surface SPD generates abundant immobile dislocations and increases material deformation resistance, which impedes continuous plastic flow and further strengthening, thereby limiting the final depth of the surface hardened layer [16]. To overcome the disadvantages of USRP, Wang et al. [29] integrated the electropulsing technique with the conventional USRP and proposed a novel EP-USRP technique, which was further applied to ameliorate the surface mechanical properties of austenitic stainless steel. The employment of electropulsing treatment contributes to the healing of surface cracks, the reduction in surface roughness, and the enhancement of the through-thickness microhardness gradient distribution in the surface-strengthened layer. Wang et al. [30] adopted EP-USRP on Ti6Al4V titanium alloy via EP-USRP, finding that optimized electric pulses could effectively facilitate surface crack healing and improving both microhardness and wear resistance. Gao et al. [31] strengthened carburized 9310 steel by EP-USRP, which greatly reduced surface roughness, remarkably increased hardness, and lowered wear loss and friction coefficient to 11.8% and 43.0% of the initial values. Qu et al. [32] achieved surface nanocrystallization on titanium alloy Ti5Al4Mo6V2Nb1Fe by EP-USRP, which remarkably improved its wear resistance.

However, existing studies on EP-USRP for improving the friction and wear performance of AA 2024-T3 are still limited, and the corresponding influence mechanism has not been fully revealed. In this work, EP-USRP was applied to strengthen the surface of AA 2024-T3. The effects of pulsed current density variations on the surface morphology, surface roughness, microhardness, and friction and wear performance were systematically investigated. The findings show that controlling and matching the pulsed current density parameters appropriately can obtain a surface-strengthened layer that has improved surface quality, increased microhardness, reduced friction coefficient and better wear resistance. In the meantime, with the different normal load cases, the difference in wear mechanisms of USRP, EP-USRP and FM samples was further explored and analyzed.

## 2. Materials and Methods

### 2.1. Materials

In this study, AA 2024-T3 was selected as the research material. As a heat-treatable Al-Cu-Mg series alloy, its detailed chemical composition is listed in Table 1. The machined workpiece was denoted as the FM sample, which was subjected to precision face milling to achieve uniform surface quality before the strengthening treatments. The FM sample was used as the original substrate in both USRP and EP-USRP experiments. Subsequently, surface strengthening treatments were carried out using USRP and EP-USRP respectively, and the corresponding treated samples were prepared for further analysis.

### 2.2. EP-USRP

**Figure 1:**
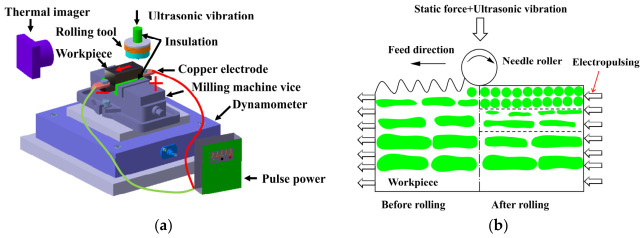
(**a**) EP-USRP platform. (**b**) Operating principle of EP-USRP.

The workpiece was tightly clamped in a precision machine vise. To ensure reliable electrical insulation of the vise, zirconia ceramic plates were placed between the vise jaws and the front/rear sides of the workpiece, as well as at the bottom of the workpiece. Additionally, a polytetrafluoroethylene (PTFE) insulating sleeve was made to cover the clamping section of the rolling tool, thereby ensuring effective insulation between the ultrasonic tool holder and the machine spindle. The USRP and EP-USRP experiments were performed on the Makino S56 vertical machining center (Makino Milling Machine Co., Ltd., Ueda, Japan). A three-axis dynamometer, Kistler 9139AA (Kistler Instrumente AG, Winterthur, Switzerland), was utilized to monitor rolling force in situ. The EP-USRP platform’s schematic diagram and operating principle are presented in Figure 1a and Figure 1b, respectively. During EP-USRP, high-energy electric pulses pass through the sample for an extremely short duration, during which the sample surface is simultaneously subjected to the coupled effects of ultrasonic vibration, rolling force, and pulse current. Ultrasonic vibration and pulsed current can both contribute effectively to the activation and motion of dislocations. Peierls stress (*τ_p_*) represents the barrier to dislocation slip and is intrinsically associated with both the material properties and the corresponding slip plane [33]:

(1) $$\tau_{p}=\frac{2\mu }{1-\upsilon }exp(-\frac{2\pi }{1-\upsilon }\frac{d}{b})$$

where *μ* is the shear modulus, *ν* is Poisson’s ratio, *b* is the Burgers vector, and *d* is the spacing of the slip plane. When ultrasonic vibration is applied, dislocations absorb acoustic energy and can more easily overcome the Peierls barrier, resulting in a significant reduction in the Peierls stress and enabling dislocation slip at a lower external load [34,35]. When an electric current passes through a metal, the generated Joule heat increases the local temperature, which lowers the yield strength and work-hardening rate and promotes dislocation motion, thus significantly softening the material and reducing the flow stress [36,37].

In this study, the electroplastic equipment is driven by a TJMC-I pulse power supply, which outputs pulse currents with an operating voltage of 60–120 V and a pulse frequency of 100–800 Hz. The positive and negative electrodes of the power supply are connected to both ends of the workpiece to form a closed conductive loop. Real-time monitoring of current signals is performed using an oscilloscope. According to the technical specifications of the pulse power supply, the relationship between the output peak pulse current (A) and the peak-to-peak voltage (V) measured by the oscilloscope is described as follows:

(2) I=V×800

where *I* is the peak pulse current, and *V* is the peak-to-peak voltage. The amplitude current density (A/mm^2^) passing through the workpiece is calculated from the peak pulse current (A) and the corresponding cross-sectional area (mm^2^), as expressed below:

(3) J=I/S

where *J* is the amplitude current density, and *S* is the cross-sectional area of the workpiece. The pulse frequency of the electroplastic equipment was fixed at 200 Hz, and the output voltage was set to 60 V, 70 V, and 80 V, respectively. When the voltage was applied to the workpiece, the peak-to-peak voltages measured by the oscilloscope were 2.48 V, 2.72 V, and 2.84 V. Meanwhile, the cross-sectional area of the workpiece is 1600 mm^2^. The peak current and amplitude current density can be calculated by substituting the measured voltages into Equations (2) and (3). The electric pulse parameters used in EP-USRP are listed in Table 2.

**Figure 2:**
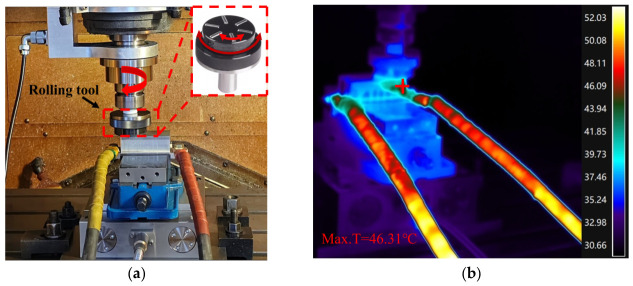
(**a**) On-site processing. (**b**) Temperature monitoring of EP-USRP sample.

The ultrasonic rolling device was operated at 19 kHz with a vibration amplitude of 3 μm. The rolling tool consists of a rolling needle, a cage, a threaded cap, and a mandrel, as shown in Figure 2a. The rolling needle is capable of rotating freely about its own axis while revolving around the cage axis. Fabricated from high-speed steel, the rolling needle has a hardness of 65 HRC and a surface roughness of Ra 0.1 μm. The rolling needle dimensions are Φ5 mm × 10 mm, with a total of 6 needles uniformly distributed along the circumference, and the circumscribed circle diameter is 50 mm. The rolling parameters were kept identical for both USRP and EP-USRP treatments with a spindle speed of 1000 rpm, a feed rate of 100 mm/min, a static pressure of 500 N, and 5 rolling passes. A mixture of engine oil and kerosene with a volume ratio of 3:7 was used for cooling and lubrication during processing. The temperature of the rolling zone was monitored in situ using a FLIR TG165-X infrared thermal imager (Wilsonville, OR, USA), as shown in Figure 2b.

### 2.3. Characterization

#### 2.3.1. X-Ray Diffractometer

Phase transformations of the samples under different processing conditions were analyzed using a Rigaku SmartLab SE X-ray diffractometer (XRD, Tokyo, Japan) with a Cu target radiation source. The scanning angle range was set to 10–80° and the scanning speed was 2°/min.

#### 2.3.2. White Light Interferometer

The surface morphology and roughness of the samples were characterized using a Contour GT-X white light interferometer (Bruker, Billerica, MA, USA), with all analyses conducted using Vision64 v5.60 software.

#### 2.3.3. Microhardness Tests

The hardness gradient testing plane was defined as the front end face of the workpiece. Workpieces treated by FM, USRP, and EP-USRP were cut into 10 × 10 × 10 mm^3^ samples using wire electrical discharge machining (WEDM). The samples were subsequently mounted with a metallographic cold-mounting compound, ground and polished using an automatic grinding and polishing machine, and ultrasonically cleaned in alcohol for 10 min. Microhardness testing was performed using an HVS-1000XYZ fully automatic micro Vickers hardness tester (Suzhou, China). Measurements were taken sequentially from the sample surface along the depth direction at 50 μm intervals for gradient characterization. The indenter was loaded to 245 mN and maintained for 10 s. Triplicate measurements were conducted at each depth.

#### 2.3.4. Friction and Wear Tests

To evaluate the friction and wear performance of all the samples, reciprocating ball-on-flat tests under dry friction conditions were conducted. All the experiments were performed using a UMT TriboLab tribometer (Bruker, Campbell, CA, USA) at room temperature of 25 °C and ambient relative humidity of 40–43%. A GCr15 steel ball with a diameter of 8 mm and hardness of 62–65 HRC was used as the counter body. The reciprocating frequency of the steel ball was 3 Hz, the unidirectional sliding stroke was 5 mm, and the total sliding duration for each sample was 1800 s. To investigate the effect of normal load on frictional behavior, normal loads of 2 N, 5 N and 8 N were selected. Before and after each test, both the steel balls and samples were ultrasonically cleaned in absolute ethanol for 10 min. A Bruker 3D white-light interferometer was utilized to characterize the morphology of the wear scars and quantify the wear volume on the sample surface. The volumetric wear rate, denoted as *W*, was subsequently calculated according to Equation (4).

(4) $$W=\frac{V}{Pvt}$$

where *W* is the volumetric wear rate (mm^3^/(N·m)), *V* is the wear volume (mm^3^), *P* is the normal load (N), *v* is the sliding velocity (m/s), and *t* is the sliding time (s).

#### 2.3.5. Scanning Electron Microscope

To elucidate the mechanisms underlying friction reduction and wear resistance, the morphology of wear scars on the sample surfaces was characterized using a CLARA ultra-high-resolution scanning electron microscope (SEM, TESCAN, Brno, Czech Republic).

#### 2.3.6. Energy Dispersive Spectroscopy

The relative contents of various elements on the worn surface were analyzed using an Xplore 30 energy-dispersive X-ray spectroscopy (EDS) detector (Oxford Instruments, Abingdon, UK).

All the quantitative experiments in this study were repeated three times. All the quantitative data, including surface roughness, microhardness, average friction coefficient, wear volume and wear rate, are presented as mean ± standard deviation (SD, *n* = 3). Error bars in all the figures indicate SD.

## 3. Results

### 3.1. XRD Phase Analysis

**Figure 3:**
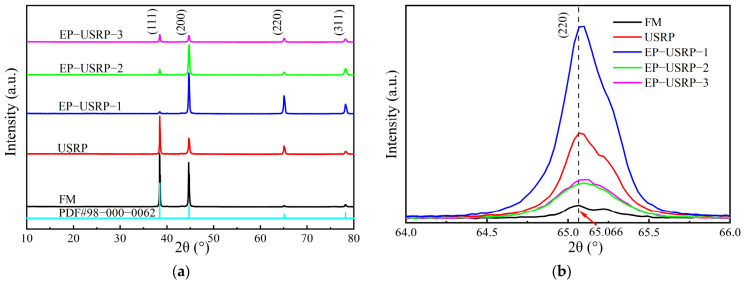
(**a**) XRD patterns of samples under different treatments. (**b**) Enlarged diffraction patterns of the (220) crystal plane for samples.

Figure 3 displays the XRD patterns of the FM, USRP, EP-USRP-1, EP-USRP-2, and EP-USRP-3 samples. As shown in Figure 3a, four distinct diffraction peaks are observed in all the samples. After phase identification using the Jade 9 software, these four main peaks are assigned to the (111), (200), (220), and (311) crystal plane diffraction peaks, in accordance with the standard PDF card No. 98-000-0062 for aluminum. Owing to the low temperature rise in the rolled regions induced by electric pulses, no oxide phases were detected in any of the EP-USRP samples. In addition, obvious differences in the intensities of the characteristic diffraction peaks can be observed between the FM sample and the other processed samples. The peak intensity variation in the FM sample is consistent with that in the standard PDF card. Compared to the FM sample, the (111) and (200) diffraction peaks of the USRP and EP-USRP samples are weakened, whereas the (220) and (311) diffraction peaks are enhanced. This indicates that preferred orientation along the (220) and (311) crystal planes is formed after USRP and EP-USRP treatments.

As shown in Figure 3b, the (220) diffraction peaks of the USRP and EP-USRP samples are broadened and shift toward higher 2θ angles due to SPD compared with the FM sample. This indicates that EP-USRP treatment may induce grain refinement and lattice distortion.

### 3.2. Surface Morphology

**Figure 4:**
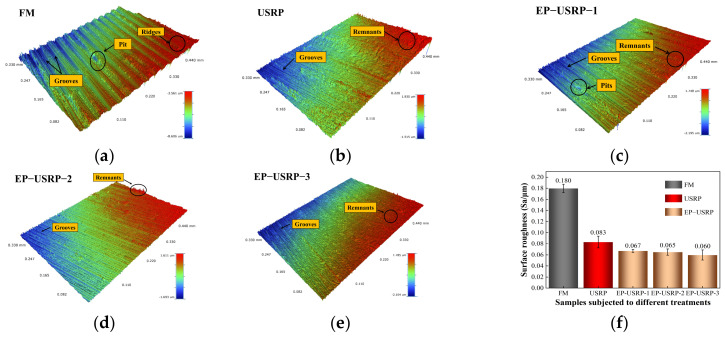
Surface roughness and evolution of micromorphology of samples subjected to different treatments: (**a**) FM, (**b**) USRP, (**c**) EP-USRP-1, (**d**) EP-USRP-2, (**e**) EP-USRP-3, (**f**) surface roughness.

Figure 4 presents the surface morphologies and corresponding surface roughness of the samples under different treatments. As shown in Figure 4a, the FM sample displays typical periodic milling textures, characterized by alternating grooves and ridges. Such a morphology is prone to inducing stress concentration and leading to a rapid increase in local stress, thereby facilitating the initiation of fatigue cracks and surface spalling. The inherent defects, such as pits and grooves on the FM sample surface, not only degrade the surface quality but also significantly reduce the wear resistance through the stress concentration effect. As a result, the FM sample is more susceptible to wear and spalling failure during friction. As shown in Figure 4b, under the coupled action of static pressure and shear stress exerted by the rolling tool, both the depth and width of the original grooves on the USRP sample surface are significantly reduced, and the ridges are extruded into the grooves by means of shear deformation, leading to a substantial improvement in surface quality.

When pulsed current is introduced during EP-USRP (Figure 4c–e), based on dislocation dynamics theory, the glide velocity of dislocations is determined by the applied mechanical stress. Under the action of an electric current, drifting electrons exert a mechanical force *F_e_* per unit length of dislocation in the same direction, as expressed in Equation (5) [38].

(5) $$F_{e}=(\frac{\Delta^{2}bV_{d}}{4V})(\frac{V_{e}}{V_{d}}-1)(\frac{\partial n_{0}}{\partial \mu })$$

where *b* is the Burgers vector, *V_e_* is the drift velocity of the electrons, *V_d_* is the speed of dislocations, *V* is the speed of the electrons in the Fermi surface, *n*_0_ is the density of the free carrier, μ is the chemical potential, and Δ is the deformation potential constant. According to Equation (5), when *V_e_* > *V_d_*, the force of free electrons is normal, thus accelerating dislocation motion. With increasing pulsed current density, the dislocation velocity is further increased, leading to a significant reduction in the flow stress of the alloy. Consequently, the plastic deformation degree on the sample surface is remarkably enhanced and more uniformly distributed, while the size of surface defects such as grooves is further reduced. This phenomenon indicates that the electroplastic effect (EPE) of the material is significantly strengthened with increasing pulsed current, thereby promoting the generation of a thicker plastic deformation layer.

As shown in Figure 4f, the surface roughness of the sample is reduced to Sa 0.083 μm due to the peak-cutting and valley-filling effects of USRP, representing a 54% reduction compared with the FM sample. After applying EP-USRP, the surface roughness is further improved. As the current density increases, the surface roughness decreases gradually. At a current density of 1.42 A/mm^2^, the surface roughness reaches a minimum value of Sa 0.060 μm.

### 3.3. Microhardness

**Figure 5:**
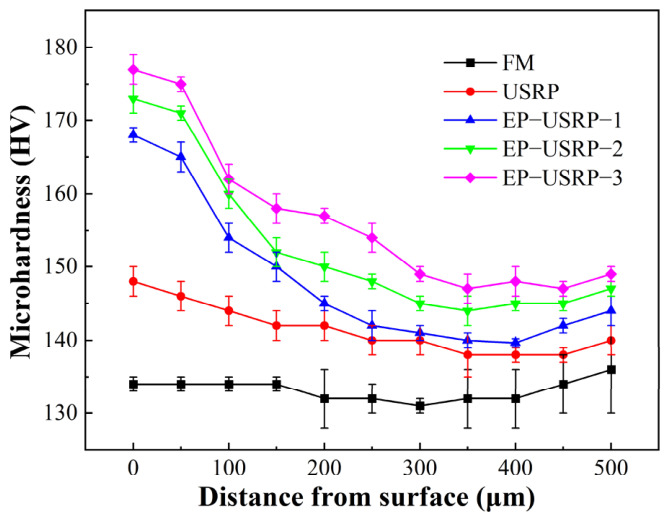
Microhardness gradient distribution of samples under different treatments.

Figure 5 shows the depth-dependent surface microhardness profiles of samples under different treatments. As observed, the microhardness of the FM sample is 134 HV, while that of the USRP sample increases to 148 HV. The surface microhardness of the EP-USRP samples is higher than that of the USRP sample. Notably, the EP-USRP-3 sample attains a maximum surface microhardness of 177 HV, corresponding to a 19.6% increase relative to the USRP sample. As the pulsed current density increases, the flow stress of the alloy decreases, thereby facilitating dislocation motion. This leads to more substantial plastic deformation in the surface layer compared with the USRP-only treatment. These findings suggest that EP-USRP can effectively strengthen the surface hardening effect and increase the thickness of the hardened layer in AA 2024-T3.

### 3.4. Friction and Wear Performance Analysis

**Figure 6:**
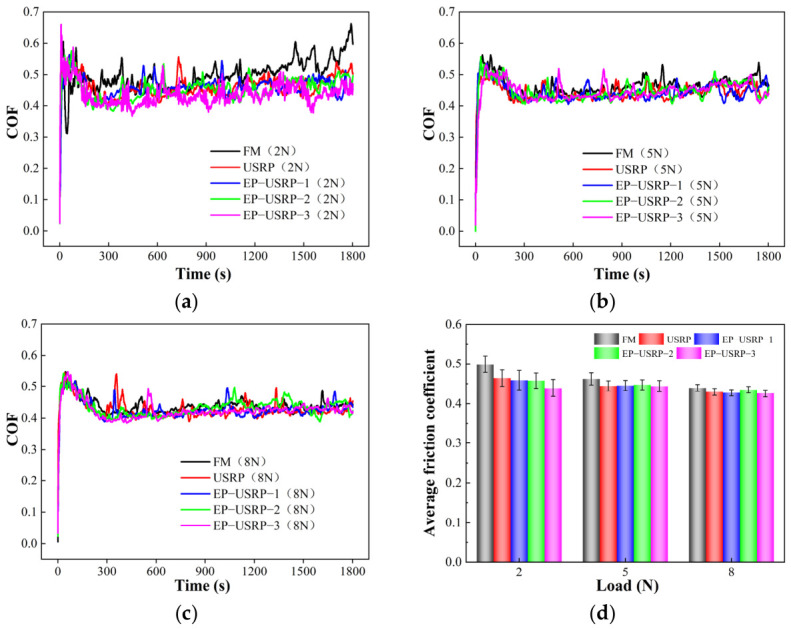
COF curves of samples subjected to different treatments under various normal loads: (**a**) 2 N, (**b**) 5 N, (**c**) 8 N, (**d**) average friction coefficient.

Figure 6a–c display the coefficients of friction (COF) of samples subjected to different treatments at normal loads of 2 N, 5 N, and 8 N, respectively. As depicted in Figure 6, the transient evolution of the friction coefficient for all the samples can be categorized into a running-in stage and a steady-state wear stage. During the running-in stage, the COF of each sample rises rapidly at the onset of sliding. This behavior is ascribed to the small initial contact area between the sample surface and the counterpart ball during the early wear phase, leading to high friction forces and thus elevated COF. After a period of sliding, the asperities on the sample surface undergo plastic deformation and shearing, which enlarges the real contact area between the mating surfaces. Therefore, the friction force decreases, causing the COF to decline gradually and stabilize eventually.

Figure 6d presents the average coefficient of friction under varying loads and different treatments. Based on adhesion theory, the friction force *F* can be divided into the shear resistance *F_b_* and plowing resistance *F_v_*, as expressed in Equation (6) [39].

(6) $$F={\;F}_{b}+F_{v}={\;A}_{r}\tau_{b}+{\;A}_{v}\sigma_{b}$$

In the equation, *A_r_* is the actual contact area, *τ_b_* is the shear strength limit of the alloy, *A_v_* is the horizontal projected area of the contact asperities, and *σ_b_* is the compressive yield strength of the material. From Equation (6), the reduction in *A_r_* and *A_v_* results in a decrease in the friction force. Both the USRP and EP-USRP samples exhibit lower surface roughness than the FM sample. The lower surface roughness corresponds to fewer micro-asperities on the sample surface, giving rise to smaller values of *A_r_* and *A_v_*. This weakens the mechanical interlocking between the counterpart ball and the micro-asperities on the sample surface, thereby reducing both shear resistance and plowing resistance, thus ultimately lowering the friction force. Both USRP and EP-USRP samples exhibit higher surface microhardness than the FM sample, making their surfaces less prone to plastic deformation under external loading. As a result, this leads to lower motion resistance during sliding friction and wear [39]. Overall, the reduced COF of the USRP and EP-USRP samples can be attributed to the lower surface roughness and enhanced surface microhardness. Furthermore, compared to USRP, the EP-USRP sample can achieve a lower COF by adjusting the pulse current magnitude.

At normal loads of 2 N, 5 N, and 8 N, the average friction coefficient of the EP-USRP-3 sample decreased by 12.00%, 3.90%, and 3.17% in comparison with the FM sample, and by 5.34%, 0.15%, and 0.88% compared with the USRP sample, respectively. Therefore, the EP-USRP-3 sample achieved the most favorable friction-reducing effect at a normal load of 2 N. In contrast, the average friction coefficients of the EP-USRP samples were comparable at 5 N and 8 N, indicating that the friction-reducing effect of EP-USRP on the sample surface was not significant within this load range.

**Figure 7:**
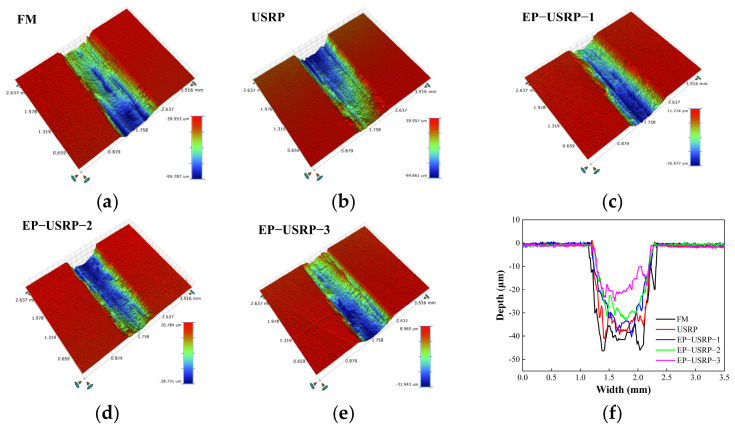
Three-dimensional morphology and cross-section profile curves of wear scars on samples with different treatments under 2 N normal load: (**a**) FM, (**b**) USRP, (**c**) EP-USRP-1, (**d**) EP-USRP-2, (**e**) EP-USRP-3, (**f**) cross-section profile curves of wear scars.

Figure 7 shows the three-dimensional (3D) morphologies and cross-sectional profiles of wear scars for the samples subjected to different treatments under 2 N. It is evident that the FM sample exhibits the maximum wear width and depth. By comparison, the wear width and depth of the USRP sample are lower than those of the FM sample. The wear width and depth are further diminished following EP-USRP, and they continue to decrease with increasing pulse current density. It is worth noting that the EP-USRP-3 sample demonstrates the minimum wear width and depth among all the samples.

**Figure 8:**
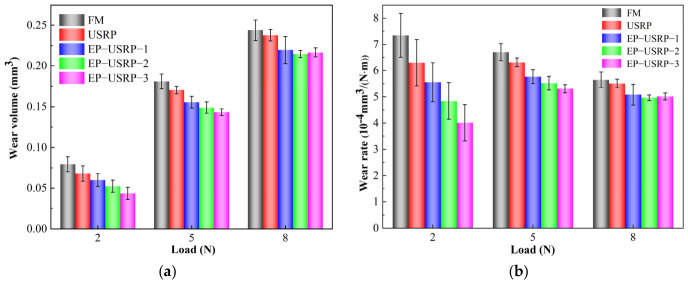
Wear volume and wear rate of FM, USRP, EP-USRP-1, EP-USRP-2 and EP-USRP-3 samples under 2 N, 5 N and 8 N: (**a**) wear volume, (**b**) wear rate.

As shown in Figure 8a, the EP-USRP-3 sample presents the lower wear volume under 2 N and 5 N, with reductions of 45.38% and 20.81%, respectively, compared to the FM sample, and 36.28% and 15.85%, respectively, compared to the USRP sample. At a normal load of 8 N, the EP-USRP-2 sample shows the minimum wear volume, with reductions of 12.02% and 9.80% compared to the FM and USRP samples, respectively.

As shown in Figure 8b, the wear rate of each sample initially increases and subsequently decreases with rising normal load. At normal loads of 2 N and 5 N, the EP-USRP-3 sample shows the lowest wear rate, corresponding to reductions of 45.37%, 36.27% and 20.81%, 15.85% compared with the FM and USRP samples, respectively. At an 8 N load, the EP-USRP-2 sample shows the lowest wear rate, with decreases of 12.02% and 9.80% compared with the FM and USRP samples, respectively. Therefore, the EP-USRP-3 sample demonstrates the optimal wear resistance under 2 N and 5 N loads, whereas EP-USRP-2 exhibits superior tribological performance at 8 N. This difference may be attributed to the fact that under high loads, the hardened layer of EP-USRP-3 possesses higher surface hardness but relatively greater brittleness in comparison with EP-USRP-2. Such material characteristics could facilitate the occurrence of three-body wear during the friction process, which is likely to result in a relatively higher wear rate of EP-USRP-3 samples [40].

## 4. Discussion

**Figure 9:**
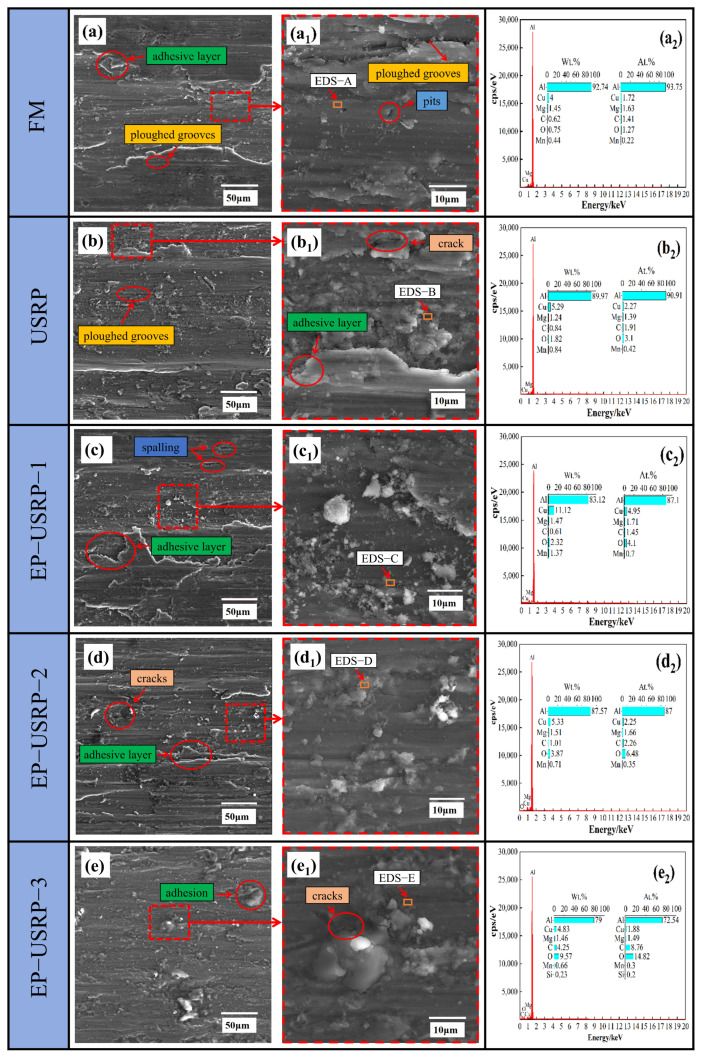
SEM morphologies of wear scars and their corresponding micro-area EDS spectra for the samples with different treatment processes at a normal load of 2 N: (**a**) FM, (**a_1_**) magnified view of FM, (**a_2_**) EDS pattern of FM, (**b**) USRP, (**b_1_**) magnified view of USRP, (**b_2_**) EDS pattern of USRP, (**c**) EP-USRP-1, (**c_1_**) magnified view of EP-USRP-1, (**c_2_**) EDS pattern of EP-USRP-1, (**d**) EP-USRP-2, (**d_1_**) magnified view of EP-USRP-2, (**d_2_**) EDS pattern of EP-USRP-2, (**e**) EP-USRP-3, (**e_1_**) magnified view of EP-USRP-3, (**e_2_**) EDS pattern of EP-USRP-3.

Figure 9 illustrates the SEM morphologies of the wear scars and the corresponding EDS spectra of selected microregions for the samples subjected to different treatments under 2 N. Distinct plowing grooves are observed in Figure 9a,a_1_. The GCr15 counterpart ball used in this test exhibits significantly higher hardness than AA 2024-T3. Thus, hard particles on the ball surface and wear debris formed during friction exert a cutting and plowing effect on the sample surface, causing the formation of plowing grooves. This result indicates that the FM sample undergoes severe abrasive wear. The pits visible in Figure 9a_1_ also arise from the action of abrasive particles. In addition, a portion of the wear debris generated on the FM sample during sliding transfers to the counterpart ball, detaches during subsequent friction, and re-adheres to the worn surface after repeated rolling and deformation, forming an adhesive layer as shown in Figure 9a. Meanwhile, a small amount of carbon from the counterpart ball also transfers to the sample surface. This phenomenon is further corroborated by the EDS spectrum at point A in Figure 9a_2_, verifying the occurrence of adhesive wear. Overall, the wear mechanism of the FM sample is mainly attributed to the combined action of abrasive and adhesive wear, which is in line with previous literature results [41].

As illustrated in Figure 9b, typical plowing morphologies are still observable on the USRP sample surface, verifying the existence of abrasive wear behavior. Nonetheless, the degree of such abrasive wear is mitigated. The USRP treatment can effectively restrain the nucleation of abrasive debris and alleviate three-body wear at the early friction stage, consequently providing the USRP sample with a lower friction coefficient, wear volume and wear rate relative to the FM sample [40]. Combined with the locally magnified image in Figure 9b_1_, distinct adhesive wear traces can also be identified on the USRP sample surface. During friction, local adhesion occurs between the sample and the counterpart ball. With continuous relative motion, wear debris accumulates on the sample surface and forms an adhesive layer containing a small amount of element C derived from the counterpart ball (Figure 9b_2_). The cracks observed in Figure 9b_1_ indicate that microcracks gradually initiate within the adhesive layer under cyclic stress. As friction proceeds, these cracks propagate continuously and eventually induce material spalling once they reach a critical stress level. Accordingly, the wear characteristics of the USRP samples are primarily characterized by abrasive wear, adhesive wear and material spalling.

Figure 9c,c_1_, 9d,d_1_, and 9e,e_1_ display the worn surface morphologies of the EP-USRP-1, EP-USRP-2, and EP-USRP-3 samples, respectively. The surfaces of the EP-USRP samples show evidence of wear debris adhesion, material spalling, and crack initiation, indicating the occurrence of adhesive wear accompanied by material peeling. The EDS spectrum at point E in Figure 9e_2_ reveals a pronounced increase in carbon content on the EP-USRP-3 sample surface, verifying the transfer of carbon from the counterpart ball to the sample surface, which is a characteristic feature of adhesive wear. Meanwhile, a high oxygen content is also observed in the spectrum, suggesting that the frictional heat promotes the oxidation reaction between the sample surface and the ambient air, thereby forming a protective oxide film. Once the oxide film becomes brittle and fractures, it generates secondary abrasive particles that exacerbate abrasive wear and alter the progression of adhesive wear. On the whole, adhesive wear, oxidative wear and material spalling are the main wear behaviors observed for the EP-USRP samples.

**Figure 10:**
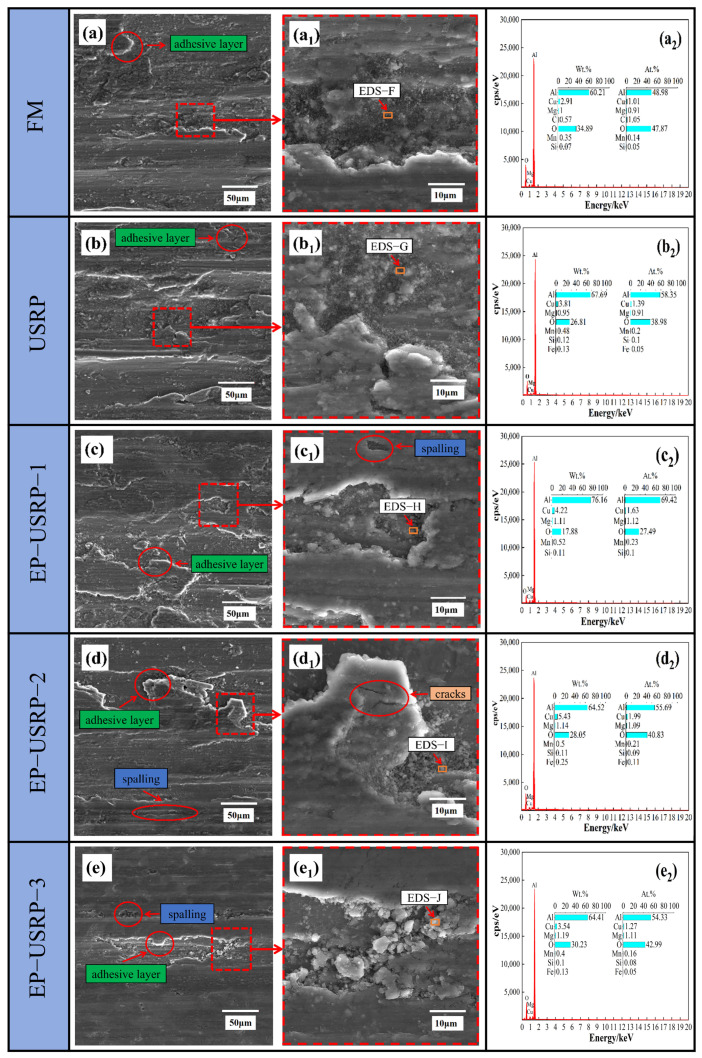
SEM morphologies of wear scars and their corresponding micro-area EDS spectra for the samples with different treatment processes at a normal load of 8 N: (**a**) FM, (**a_1_**) magnified view of FM, (**a_2_**) EDS pattern of FM, (**b**) USRP, (**b_1_**) magnified view of USRP, (**b_2_**) EDS pattern of USRP, (**c**) EP-USRP-1, (**c_1_**) magnified view of EP-USRP-1, (**c_2_**) EDS pattern of EP-USRP-1, (**d**) EP-USRP-2, (**d_1_**) magnified view of EP-USRP-2, (**d_2_**) EDS pattern of EP-USRP-2, (**e**) EP-USRP-3, (**e_1_**) magnified view of EP-USRP-3, (**e_2_**) EDS pattern of EP-USRP-3.

Figure 10 illustrates the SEM morphologies of the wear scars and the corresponding EDS spectra of selected microregions for the samples under different treatments under 8 N. As depicted in Figure 10a, obvious adhesive layers are present on the FM sample surface. During sliding, wear debris accumulates continuously at the contact interface, thereby forming an adhesive layer. As shown in Figure 10a_2_, the oxygen content at point F on the wear debris is 34.89 wt.% and 47.87 at.%, respectively, confirming that oxidation occurred on the sample surface during testing. Overall, adhesive wear is the predominant mechanism in the wear process of the FM sample. Furthermore, the frictional heat promotes the oxidation of wear debris, indicating that adhesion and oxidation act synergistically to accelerate the material removal process.

As shown in Figure 10b, the wear morphology of the USRP sample is analogous to that of the FM sample. The oxygen content at Point G on the wear debris is 26.81 wt.% and 38.98 at.%, respectively. This result indicates that the wear mechanism of the USRP samples is mainly dominated by adhesive wear, together with oxidative wear.

As shown in Figure 10c,c_1_, 10d,d_1_ and 10e,e_1_, adhesive layers are observed on all the worn surfaces of the EP-USRP samples, respectively, indicating that adhesive wear is the predominant wear mechanism. Interestingly, all the worn surfaces become progressively smoother with increasing pulse current compared with those of the FM and USRP samples. This can be attributed to the higher surface microhardness of the EP-USRP samples, which facilitates brittle fracture and spalling of the surface adhesive layers during friction. Consequently, the wear mechanism is governed by fatigue wear. This observation indicates that the applied pulse current can tailor the surface microstructure and surface properties through the EPE, thereby regulating adhesion, crack propagation, and spalling behavior, and ultimately controlling the wear mechanism. Furthermore, as presented in Figure 10e_2_, the oxygen content at point J on the wear debris of the EP-USRP-3 sample is 30.23 wt.% and 42.99 at.%, respectively, which confirms that the wear mechanism is accompanied by oxidative wear. In summary, under 8 N, the wear mechanisms of the FM and USRP samples are primarily characterized by adhesive wear and oxidative wear. In contrast, the EP-USRP samples show a combined wear mechanism involving adhesive wear, oxidative wear and material spalling. The similarity in wear mechanisms among the samples with different treatments accounts well for the relatively close average friction coefficients under this load condition.

## 5. Conclusions

In this paper, a systematic investigation was conducted to examine the effects of EP-USRP on the friction and wear properties of AA 2024-T3. Based on analyses of the surface morphology, mechanical properties, and tribological behavior of the treated samples, the main conclusions can be summarized as follows:

(1) EP-USRP treatment can effectively lower the flow stress of the alloy and promote its plastic deformation capability. Meanwhile, the surface hardness is increased while the surface roughness is decreased. Among all the samples, the EP-USRP-3 sample treated at a current density of 1.42 A/mm^2^ achieved 27.7% lower surface roughness and 19.6% higher microhardness compared with the USRP sample.
(2) EP-USRP treatment improves the tribological performance of AA 2024-T3, generally outperforming both the USRP and FM samples. EP-USRP-3 exhibits the best anti-friction and anti-wear performance at 2 N, with reductions of 5.34% in average friction coefficient, 36.38% in wear volume, and 36.27% in wear rate compared to the USRP sample. At 8 N, however, EP-USRP-2 shows the lowest wear volume and wear rate. These findings confirm that the synergistic effect of electropulsing and ultrasonic surface rolling effectively enhances the tribological properties of AA 2024-T3, with the optimal processing parameters varying with the normal load.
(3) EP-USRP treatment may alter the wear characteristics of AA 2024-T3 alloy, showing differences from the USRP and FM samples. The EP-USRP samples tend to display adhesive wear, oxidative wear and material spalling. In comparison, the USRP specimens exhibit adhesive wear, abrasive wear, and oxidative wear together with material spalling, while FM samples are mostly featured with adhesive wear, abrasive wear and oxidative wear. The reduced degree of abrasive wear in EP-USRP samples is likely to contribute to their improved tribological properties.
(4) Appropriately adjusting pulsed current density in the EP-USRP may modify the properties of the surface-strengthened layer of AA 2024-T3. This modification is conducive to obtaining a strengthened layer with enhanced surface mechanical performance and preferred friction and wear resistance.

## Figures and Tables

**Table 1:** Chemical composition of AA 2024-T3 (wt.%).

Chemical Element	Si	Fe	Cu	Mn	Mg	Ni	Zn	Ti	Al
Content	0–0.50	0–0.50	3.80–4.90	0.30–0.90	1.20–1.80	0–0.10	0–0.30	0–0.15	Balance

**Table 2:** Electropulsing parameters in EP-USRP.

Samples	Frequency (Hz)	Peak-to-Peak Voltage (V)	Peak Pulse Current (A)	Amplitude Current Density (A/mm^2^)	Duration (μs)	Temperature (°C)
FM	-	-	-	-	-	25
USRP	-	-	-	-	-	25
EP-USRP-1	200	2.48	1984	1.24	100	40.49
EP-USRP-2	200	2.72	2176	1.36	100	43.01
EP-USRP-3	200	2.84	2274	1.42	100	46.31

## Data Availability

The data presented in this study are available upon request from the corresponding author. The data are not publicly available due to privacy and ongoing research.
